# Prenatal exposure to antifungal medication may change anogenital distance in male offspring: a preliminary study

**DOI:** 10.1186/s12940-017-0263-z

**Published:** 2017-06-21

**Authors:** Djamilla Madelung Mogensen, Maria Bergkvist Pihl, Niels E. Skakkebæk, Helle Raun Andersen, Anders Juul, Henriette Boye Kyhl, Shanna Swan, David Møbjerg Kristensen, Marianne Skovager Andersen, Dorte Vesterholm Lind, Tina Kold Jensen

**Affiliations:** 1 Department of Environmental Medicine, Institute of Public Health, University of Southern Denmark, Winsloewsparken 17 2, 5000 Odense C, Denmark; 2 Department of Growth and Reproduction and EDMaRC, Rigshospitalet, University of Copenhagen, Copenhagen, Denmark; 3 Hans Christian Andersen Children’s Hospital, Odense University Hospital, Sdr. Boulevard 29, 5000 Odense C, Denmark; 4 Odense Patient data Exploratory Network (OPEN), Odense University Hospital, DK-5000 Odense, Denmark; 5 Department of Preventive Medicine, Icahn School of Medicine at Mount Sinai, New York, NY USA; 6 Department of Biology, Laboratory of Genomics and Molecular Biomedicine, University of Copenhagen, DK-2100 Copenhagen, Denmark; 7 Department of Endocrinology, Odense University Hospital, DK-5000 Odense, Denmark

**Keywords:** Anogenital distance, Prenatal exposure, Antifungal medicine, Endocrine disruptors

## Abstract

**Background:**

Vaginal candidiasis is frequent among pregnant women and it is treated with anti-fungal medication (conazoles). Conazoles have anti-androgenic properties and prenatal exposure in rodents is associated with a shorter (less masculine) anogenital distance (AGD) in male offspring. To our knowledge this has never been studied in humans.

**Method:**

In the Odense Child Cohort pregnant women residing in Odense municipality, Denmark, were recruited at gestational age 8–16 weeks between 2010 and 2012. Of the eligible 2421 mother-child pairs, 812 mother-son pairs were included. Questionnaire data on medicine use were collected in first and third trimester and physical examination at age 3 month was performed.

Ano-scrotal distance; measured from the centre of anus to the posterior base of scrotum (AGDas). Ano-cephalad distance; measured from the centre of anus to the cephalad insertion of the penis (AGDap) and penile width; measured at the base of the penis.

**Results:**

Eighty seven women had used antifungal medicine during pregnancy. Maternal use of oral fluconazole (*n* = 4) was associated with a 6.4 mm shorter AGDas (95% CI: -11.9;-0.9) in the male offspring. Use of antifungal vaginal tablets (*n* = 21), was associated with a non-significantly shorter AGDas (−1.9 mm; 95% CI: -4.3; 0.5) whereas exposure to vaginal cream (*n* = 23) was not associated to AGDas. Use of antifungal medicine in the window of genital development between 8 and 14 weeks of gestation was associated with a larger reduction in AGDas than exposure outside this window. Antifungal medicine intake was not associated with AGDap and penil width.

**Conclusion:**

Our preliminary findings prompted us to hypothesize that maternal use of conazole antifungal medication during pregnancy may affect the masculinization of male offspring. If confirmed, pregnant women should be advised to use antifungal medicine with caution.

**Electronic supplementary material:**

The online version of this article (doi:10.1186/s12940-017-0263-z) contains supplementary material, which is available to authorized users.

## Background

In pregnancy, the prevalence of vaginal candidiasis is increased compared to those not pregnant [1]. Treatment is antifungal compounds administered locally or systemically depending on the severity of symptoms [2]. In Denmark, antifungal vaginal tablets and antifungal vaginal cream with either the active ingredients miconazole or clotrimazole (both imidiazoles) are recommended as first treatment for vaginal candidiasis. Oral fluconazole (triazole) is only recommended for severe cases when the woman is pregnant [3–9]. Imidazole and triazole compounds are anti-mycotic through inhibition of a specific cytochrome P450 enzyme (CYP51) involved in fungal cell wall synthesis, [10, 11] but they are also known inhibitors of a range of other CYP enzymes including those involved in androgen biosynthesis [11, 12]. Accordingly, anti-androgenic properties have been demonstrated for several conazole fungicides [12, 13].

In pregnancy, hormonal changes increase the risk of vaginal candidiasis, [1, 14, 15] and as the fetus is particularly vulnerable to endocrine disruption, use of anti-fungal medication might affect development of the genitalia [16–18]. A recent Danish study among 1,405,663 pregnant women found an association between use of oral fluconazole during pregnancy and miscarriage [19]. Case reports have linked high-dose, long-term treatment with oral fluconazole during pregnancy to a pattern of skeletal and craniofacial birth defects seen in the offspring. This has raised concern regarding the safety of oral fluconazole use during pregnancy [20–23]. Other studies have examined the association between maternal use of antifungal medicine and malformations in the offspring, but no consistent associations have been found [14, 24, 25]. Subtle signs of anti-androgen action have not been studied in children whose mothers used anti-fungal medication during pregnancy.

Anogenital distance (AGD: distance from anus to genitals) is routinely used in animal toxicology studies and is a sensitive test of exposure of the male fetus to anti-androgenic agents [26–28]. In rodents, AGD has been shown to reflect the amount of androgen to which a male fetus is exposed in early development: in utero exposure to lower levels of androgens results in a shorter AGD, which is one marker of de-masculinisation [26, 28]. A vulnerable fetal masculinisation programming window (MPW) has been identified in rodent models in which androgens must act to masculinise the components of the reproductive tract and to allow the later complete development. This MPW has been identified in human to occur between gestation weeks 8–14 [29].

To our knowledge, no human studies have examined the association between maternal exposure to antifungal medicine and AGD in the male offspring. We therefore prospectively investigated the association between systemic or local use of antifungal medicine during pregnancy and the subsequent AGD and penile width in the male offspring at 3 months of age in the Odense Child Cohort (OCC) study.

## Methods

Newly pregnant women at gestational age 8–16 weeks residing in Odense Municipality, Denmark, between 2010 and 2012 were recruited: at a voluntary information meeting about ultrasound examinations; at their first antenatal midwife visit; or at their ultrasound examination at Odense University Hospital. All of the pregnant women in the study completed two questionnaires, one during the first and one during the third trimester [30]. Serum samples were collected twice and urine samples once during pregnancy and stored in freezers at the Odense Patient data Explorative Network (OPEN) [30]. The study was carried out in accordance with the Helsinki Declaration II and was approved by the Regional Scientific Ethical Committees for Southern Denmark (S-20090130).

Of the eligible population of 6707 pregnant women, 4017 women were informed about the study and 2421 live born singletons with birth register data participated in the study and are currently being followed up. Participants were better educated (high school +1 year or more) and more often of Danish origin than non-participants [30].

In the questionnaire administered during the first trimester, participants were asked whether they had taken any topical or systemic medications (both prescription and over the counter) and if so to provide the name, dose and gestational week/s of use of each named medication. In the second questionnaire (administered during the third trimester) the women were asked to state whether they had experienced specific diseases or symptoms (including vaginal candidiasis) during the last 3 months of pregnancy. Furthermore, the women were asked to state whether they had ever used any type of medication during their entire pregnancy. They were asked to note names of medication, doses, total number of days of use and in which gestational week(s) it was used.

The answers from both questionnaires were categorised into: overall use of any type of antifungal medicine during pregnancy; use of fluconazole oral tablets; use of antifungal vaginal tablets (including antifungal vaginal cream if used in combination with vaginal tablets); use of antifungal vaginal cream only; and unspecific use of antifungal medicine (women who had not specifically noted which type of antifungal medicine they had used). In addition, we categorised use of antifungal oral and vaginal tablets into exposure in and outside the MPW (gestation weeks 8–14).

Three months after the expected date of birth, regardless of actual gestational age at birth, the children were invited for a clinical examination, which included measurements of length, weight and AGD. The two measurements of AGD, and the measurement of penile width were made using a Vernier caliper, which is the recommended instrument for AGD measurement [31]. The shorter AGD measurement was measured from the centre of anus to the posterior base of scrotum (AGDas) and the longer from the centre of anus to the cephalad insertion of the penis (AGDap). Penile width was measured at the base of the penis. All of these measurements were made three times and the arithmetic mean was calculated. Expert-trained technicians performed the examinations [30]. 13 boys had AGD measured by two examiners. The coefficient of variation (CV) was 3% for all the triplicate AGD measurements. Inter-examiner CV was respectively 4%, 3% and 4% for AGDas, AGDap and penile width indicating a high degree of consistency.

### Statistical analysis

Use of antifungal medicine during pregnancy was categorized into: no use (reference group); fluconazole oral tablets; vaginal tablets; vaginal cream; and use of antifungal medicine of unspecified type. Differences in distributions of use of antifungal medicine according to population characteristics were assessed by chi-square.

Univariate associations between use of antifungal medicine during pregnancy, and AGD and penile width at 3-months of age, were examined. Multiple linear regressions were then used to adjust for potential confounding factors. AGD values vary with age and weight of the child, and because the clinical examination was scheduled to take place three months after expected date of birth we constructed a measure of ‘post-conceptional age’ defined as the sum of gestational age at birth (in days) and the age of the child at the AGD measurements (in days). Multiple regression analyses were thus adjusted for the post-conceptional age and individual weight-for-age standard deviation score (Z-score) [32]. We examined several potential confounding factors including maternal age, maternal body mass index, ethnicity, smoking, alcohol consumption as well as parity, socioeconomic status, gestational age at the 3-month examination and birth weight. None of these factors changed the estimated beta-coefficient more than 10% and were therefore excluded from the final models.

We evaluated the fit of the regression models by testing the residuals for normality and by inspecting the residual plots. All statistical analyses were conducted in STATA13. *P*-values were considered significant when they were <0.05, and 95% confidence intervals were calculated.

## Results

**Fig. 1:**
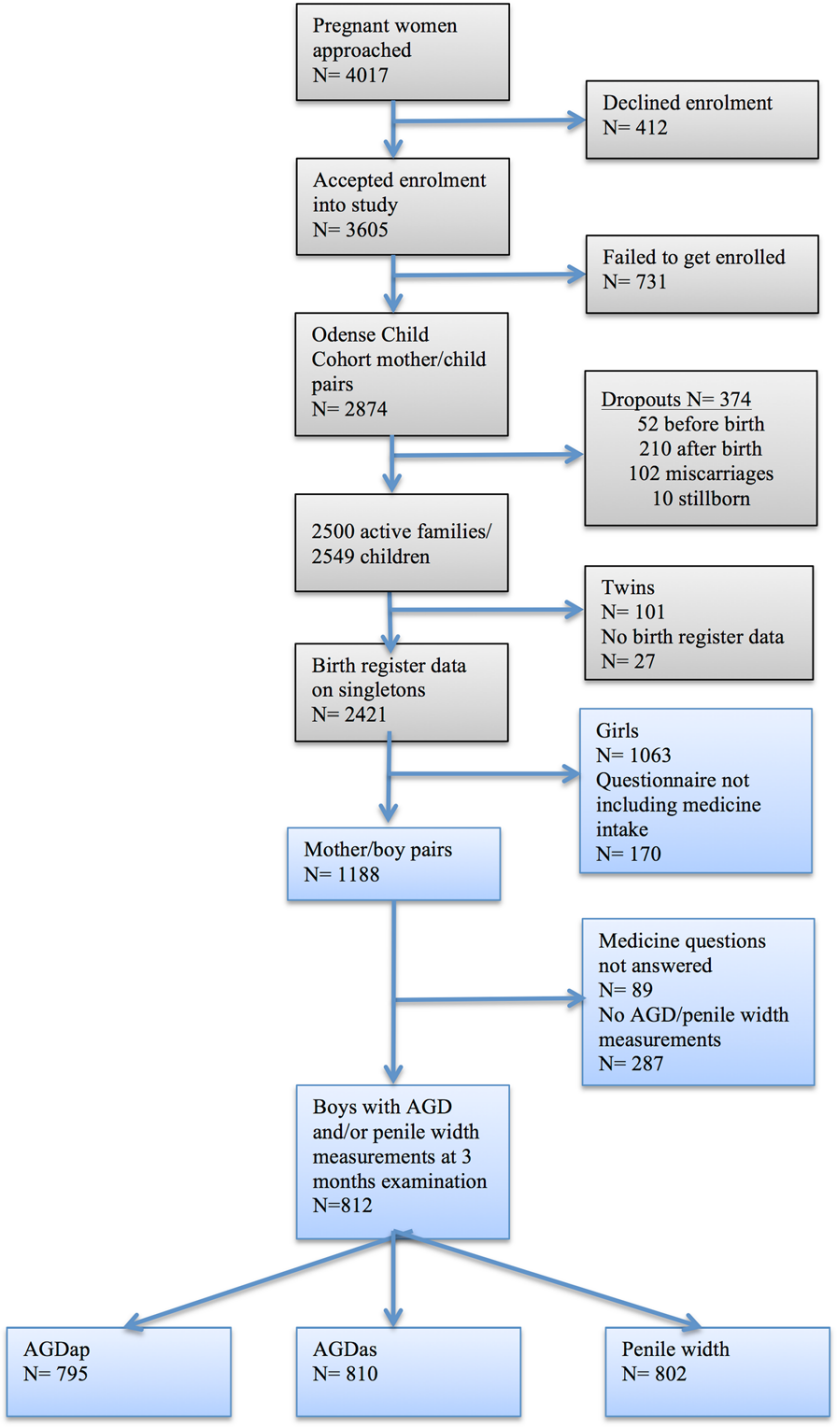
Numbers of infants included in Odense Child Cohort and in the analysis

A total of 2421 live born singletons with birth registry data were included in the original OCC dataset. After the initial exclusions, as shown in Fig. 1, the final dataset consisted of 812 mother-son pairs with measurements at 3 months of whom there were 795 AGDap measurements, 810 AGDas measurements and 802 measurements of penile width.

Pregnant women using anti-fungal medicine were more often of non-European origin and had a shorter gestational age, but there were no differences in age, parity, smoking habit, alcohol intake, BMI or birth weight (Table 1).

**Table 1:** Population characteristics of 812 mother/boy pairs included in the study according to antifungal medicine use in percent

	Population characteristics *N* = 812 (%)	No use of antifungal medicine *N* = 725	Use of some form of antifungal medicine *N* = 87
Maternal age (y)			
<25	72(9)	9	9
25–35	555(72)	71	80
>35	150(19)	20	11
BMI (kg/m^2^)			
<18.5	24(3)	3	6
18.5–24.9	495(61)	61	58
25–29.9	187(23)	23	22
> = 30	105(12)	13	14
Maternal ethnicity			
European	602(95)	96^*^	88^*^
Non-European	32(5)	4^*^	12^*^
Maternal smoking			
No	772(97)	97	97
Yes	25(3)	3	3
Alcohol consumption			
No	565(91)	92	88
Yes	54(9)	8	12
Parity			
Nulliparous	447(55)	55	53
Multiparous	364(45)	45	47
Socio economic status^a^			
Low	17(3)	2	5
Intermediate	189(30)	31	27
High	418(67)	67	68
Gestational age (weeks) at birth			
<37	28(4)	4^*^	4^*^
37–40	538(68)	66^*^	79^*^
>40	231(29)	30^*^	18^*^
Birth weight (g)			
<2500 g	18(2)	3	0
2500-3999 g	604(76)	75	79
> = 4000 g	175(22)	22	21

^*^ *p*-value < 0.05 calculated by chi-square test

^a^Low = high school or less, Intermediate = high school +1–3 years further education, High = high school +4 years or more further education

Of the overall study population of 812 mother-son pairs, 87 (11%) women had used antifungal medicine during pregnancy (Table 1) of whom 4 women had been treated with oral tablets containing fluconazole (a single dose of 150 mg) within the MPW. In addition, 21 women had used vaginal tablets containing 500 mg to 1200 mg miconazole or clotrimazole of whom 11 had used them in the MPW. Furthermore, of the 21 women who had used vaginal tablets, 11 had used only antifungal vaginal tablets and the other 10 had used them in combination with antifungal vaginal cream. There were 23 women who had only used antifungal vaginal cream (active ingredients miconazole or clotrimazole) and 39 women had used antifungal medicine but did not specify type (Table 2 and Additional file 1).

**Table 2:** Mean (SD) anogenital distance (AGDas) in mm in boys at three months according to maternal use of antifungal medicine in pregnancy. Beta values represent adjusted mean difference calculated from multiple liniar regeression (95% confidence intervals, 95% CI) in AGD in boys whose mothers used antifungal medicine in pregnancy, compared to non-users

		AGDas		
	N	Mean (SD) mm	β^a^ mm	95% CI
No antifungal medicine use (reference)	723	36.1 (5.7)	Reference	
Vaginal tablets^b^	21	35.0 (7.2)	−1.9	(−4.3; 0.5)
Only vaginal tablets^c^	11	33.5 (7.5)	−3.0	(−6.3; 0.3)
Vaginal tablets in combination with cream^c^	10	36.7 (6.8)	−0.7	(−4.1; 2.8)
Vaginal tablets *in* masculinisation programming window^d^	11	35.0 (8.1)	−2.2	(−5.5; 1.1)
Vaginal tablets *outside* the masculinisation programming window^d^	10	35.0 (6.4)	−1.6	(−5.0; 1.9)
Vaginal cream^b^ only	23	35.9 (6.7)	0.1	(−2.3; 2.4)
Fluconazole oral tablets	4	28.7 (4.2)^e^	−6.4^e^	(−11.9; −0.9)^e^
Use of antifungal medicine but type not specified	39	35.5 (6.5)	−0.9	(−2.7; 0.9)

^a^Adjusted for age and z-score for weight

^b^Active ingredients miconazole or clotrimazole

^c^Sub-analysis where antifungal vaginal tablet use (*N* = 21) has been divided into “only vaginal tablet use” and “vaginal tablet used in combination with cream”

^d^Sub-analysis where antifungal vaginal tablet use (*N* = 21) has been divided into “Vaginal tablets *in* masculinization programming window” and “Vaginal tablets *outside* the masculinization programming window”

^e^ *p*<0.05 after the adjusted oral tablets

**Fig. 2:**
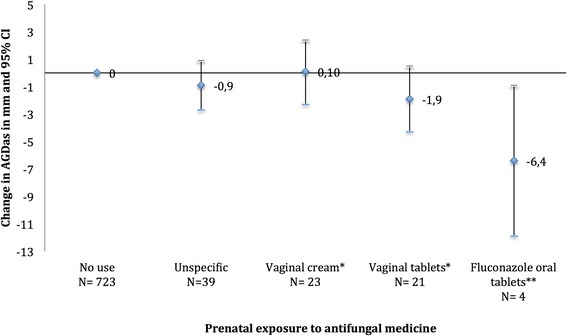
Mm reduction of AGDas (and 95% confidence intervals) according to maternal use of antifungal medicine adjusted for age and z-score for weight from multiple linear regression

**Fig. 3:**
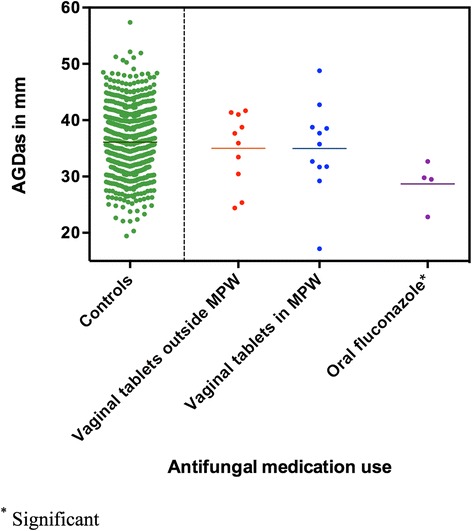
Column scatterplot of AGDas in mm among boys not prenatally exposed to antifungal medicine (green dots), boys prenatally exposed to antifungal vaginal tablets *outside* the MPW (red dots), boys prenatally exposed to antifungal vaginal tablets *in* the MPW (blue dots), and boys prenatally exposed to oral fluconazole (purple dots)

After adjustment for weight-adjusted z-score and post-conceptional age, boys prenatally exposed to oral fluconazole had a significantly shorter AGDas (−6.4 mm; 95% CI: -11.9; −0.9) at their 3-month examination compared to unexposed boys (Table 2, Figs. 2 and 3). In addition, maternal use of antifungal vaginal tablets containing miconazole or clotrimazole was marginally associated with a shorter AGDas (−1.9 mm; 95% CI: -4.3; 0.5). Boys exposed to vaginal tablets containing either miconazole or clotrimazole in the MPW had shorter AGDas than those exposed outside the MPW (Table 2). When dividing use of vaginal tablets into only use of vaginal tablets and vaginal tablet use in combination with cream, boys prenatally exposed to only vaginal tablets had shorter AGDas than those prenatally exposed to a combination (Table 2).

An association was found between unspecific antifungal medicine use and penile width (−0.5 mm; 95%CI: -0.9; −0.1) but no association was seen for penile width and other exposure groups (see Additional file 1). No association was found between prenatal exposure to any antifungal medicine and AGDap.

## Discussion

Four pregnant women taking fluconazole orally gave birth to boys with a significantly shorter AGDas (−6.4 mm) than unexposed boys. In addition, maternal use of antifungal vaginal tablets containing miconazole or clotrimazole was associated with a shorter AGDas, although not significantly, whereas use of the same compounds in the form of vaginal creams did not affect AGDas. AGDap and penile width were not affected apart from a shorter penile width among boys to mothers with unspecific use, which was probably due to chance. Interestingly, use of antifungal medicine in the window of genital development between 8 and 14 weeks of gestation was associated with a larger reduction in AGDas than exposure outside of this window. This is in accordance with the sensitivity of this window to the effects of prenatal anti-androgen exposure in rodent studies [27]. We acknowledge that our rather preliminary findings are based on four cases, but they are biologically plausible and of public health importance as up to 20% of pregnant women experience vaginal candidiasis. In addition, a 6.4 mm shorter AGD corresponds to a 20% decrease and may be of clinical importance as it has been associated with abnormal adult male reproduction [33, 34].

This is to our knowledge the first human study to examine the association between maternal exposure to antifungal medicine and AGD in the offspring. Our findings are biologically plausible, as conazole antifungals have known anti-androgenic properties, [12] and exposure to chemicals with anti-androgenic action (e.g. phthalates) have been associated with shorter AGD in several human studies [32, 35–38]. Rodent studies have shown that an impairment of androgen action within the MPW can result abnormal development and function of reproductive organs [27]. Hence, exposure to anti-androgenic chemicals, including conazole antifungals, during the MPW have resulted in hypospadias, cryptorchidism and shortened AGD in rodent studies [26, 27].

Two large registry-based Danish studies found no association between oral intake of fluconazole during the first trimester and 15 different birth defects in the offspring [24, 25], however, they did not measure AGD. Similar findings have been reported in other smaller studies [14, 15, 24], which also studied hypospadias, but none of the studies measured AGD, which may be a more sensitive marker of prenatal exposure to anti-androgens [32].

The association between maternal use of antifungal vaginal tablets and AGD was weaker than among boys exposed to oral treatment. This may be due to less potent anti-androgenic action of miconazole and clotrimazole and/or lower exposure from vaginal tablets. The same reasoning may be used for the lack of effect of vaginal cream exposure on AGDas, as exposure levels from creams, is expected to be even lower. Miconazole in vaginal tablets have been demonstrated to be absorbed systemically [39]. Women using vaginal tablets without combination with antifungal vaginal cream typically used tablets containing higher doses of miconazole or clotrimazole than women who used both tablets and cream. Thus, the fetus may be exposed to a higher dose after use of vaginal tablets alone compared to the combined treatment of vaginal tables and cream which was seen by a shorter ADGas in this group.

Participation rate was 43% [30] and only 11% of the women reported use of antifungal medicine, which is lower than the estimated 20% of all pregnant women suffering vaginal candidiasis [14, 15]. This could be due to the fact that our study population was higher educated and therefore more aware of the importance of avoiding medication use during pregnancy. Information about use of antifungal medicine was self-reported which might have led to misclassification. This is, however, not likely to be associated with AGD as the women were unaware of this measure when answering the questionnaires leading to an underestimation of the association.

AGD measurements has acceptable intra- and inter-examiner reliability, but with large inter-individual variation in measurements especially in AGDap measured from the anus to the top of the cephalad insertion of the penis. AGDap is therefore more dependent on the size of the child, introducing a higher inaccuracy of the measurement. Also, penile width is small and thus the same absolute measurement error is of relatively larger importance. Confounding by indication may explain our findings, as the overgrowth of *Candida albicans* organism may be associated with a reduction in AGD, rather than the use of antifungal medication. We adjusted for relevant confounders but we cannot exclude the possibility of residual confounding from for example co-exposure to other environmental chemicals, lifestyle or health behavior.

In male rodents, the shortened AGD persists into adulthood confirming that interference of androgenic activity permanently alters the reproductive tissues [29]. In cross-sectional studies among adult men, AGD has been shown to have a significant positive association with sperm count [34]. In fact, AGD is now considered part of the spectrum of male intrauterine testicular disruption called the testicular dysgenesis syndrome (TDS) [18]. Therefore, the observed reduction in AGD may have potential long-term consequences for male reproductive health.

## Conclusion

Based on our findings that four pregnant women taking fluconazole orally gave birth to boys with a significantly shorter AGD we hypothesize that maternal use of antifungal medication may cause anti-androgenic effects. Further studies on maternal antifungal use during pregnancy are needed. In the interim, pregnant women should be advised to use antifungal medicine with caution.

## Additional file

Additional file 1: Table S1. Use of antifungal vaginal tablets (active ingredients clotrimazole or miconazole) and oral fluconazole and in relation to anogenital distance (AGDas, AGDap) and penile width measurements among 25 mother/boy pairs. **Table S2.** Mean (SD) penile width in mm in boys at three months according to maternal use of antifungal medicine in pregnancy. Beta values represent adjusted mean difference (95% confidence intervals, 95% CI) in penile width in boys whose mothers used antifungal medicine in pregnancy, and non-users. (DOCX 110 kb)

## Abbreviations

AGDap: Anogenital distance measured from the centre of anus to the cephalic insertion of the penis
AGDas: Anogenital distance measured from the centre of anus to the posterior base of scrotum
MPW: Masculinization programming window
OCC: Odense child cohort
TDS: Testicular dysgenesis syndrome
