# Supplementary material for: Prenatal exposure to antifungal medication may change anogenital distance in male offspring: a preliminary study
Source: Environ Health. 2017 Jun 21;16:68. doi: 10.1186/s12940-017-0263-z (PMC5480178; doi:10.1186/s12940-017-0263-z)
Supplement: Additional file 1: Table S1. — Use of antifungal vaginal tablets (active ingredients clotrimazole or miconazole) and oral fluconazole and in relation to anogenital distance (AGDas, AGDap) and penile width measurements among 25 mother/boy pairs. Table S2. Mean (SD) penile width in mm in boys at three months according to maternal use of antifungal medicine in pregnancy. Beta values represent adjusted mean difference (95% confidence intervals, 95% CI) in penile width in boys whose mothers used antifungal medicine in pregnancy, and non-users. (DOCX 110 kb) [file 12940_2017_263_MOESM1_ESM.docx]

**Appendix**

Table I Use of antifungal vaginal tablets (active ingredients clotrimazole or miconazole) and oral fluconazole and in relation to anogenital distance (AGDas, AGDap) and penile width measurements among 25 mother/boy pairs.

|  | | | | | **AGDas (mm)** | **AGDap (mm)** | **Penile width (mm)** | **Days since conception** | **Birth weight (g)** | **Gestational age (weeks)**  **at birth** | **Maternal age (y)** | **Maternal BMI** |
| --- | --- | --- | --- | --- | --- | --- | --- | --- | --- | --- | --- | --- |
|  | | | | | mean | mean | Mean | mean | mean | mean | mean | mean |
| No exposure to antifungal medicine | | | |  | 36.1 | 71.2 | 13.5 | 396 | 3595 | 40 | 30 | 25 |
| **Antifungal vaginal tablet use** | | | | | | | | |  |  |  |  |
|  | **Weeks** | **Doses (mg)** | **Cream (+/-)** | | **AGDas (mm)** mean | **AGDap (mm)** mean | **Penile width (mm)** mean | **Days since conception** | **Birth weight (g)** | **Gestational age (weeks)**  **at birth** | **Maternal age (y)** | **Maternal BMI** |
| 1 | - | 1200 | - | | 35.9 | 67.8 | 12.9 | 369 | 2660 | 38 | 29 | 25 |
| 2 | 4-9  20-24 | 500  500 | - | | 38.5 | 68.8 | 13.3 | 383 | 4445 | 41 | 30 | 24 |
| 3 | 25-29 | 500 | + | | 41.4 | 77.1 | 14.3 | 367 | 3875 | 40 | 33 | 21 |
| 4 | 20-24 | 500 | - | | 24.4 | 49.1 | 13.4 | 369 | 4420 | 40 | 25 | 24 |
| 5 | 25-29 | 500 | + | | 30.5 | 87.8 | 14.6 | 378 | 4230 | 40 | 34 | 20 |
| 6 | 10-14 | 500 | + | | 31.8 | 64.4 | 12.9 | 389 | 3425 | 41 | 31 | 24 |
| 7 | 15-19 | 500 | - | | 31.7 | 71.9 | 12.3 | 383 | 3655 | 38 | 23 | 21 |
| 8 | 15-19  25-29 | 600  500 | - | | 37.7 | 77.9 | 13.2 | 413 | 3850 | 40 | 27 | 18 |
| 9 | 4 | 1200 | - | | 38.8 | 72.1 | 11.7 | 420 | 2925 | 38 | 31 | 20 |
| 10 | 20-24 | 500 | + | | 37.7 | 70.4 | 13.3 | 406 | 3175 | 40 | 28 | 20 |
| 11 | 10-14 | 1200 | - | | 42.7 | 79.0 | 14.7 | 388 | 4080 | 40 | 33 | 32 |
| 12 | 13 | 500 | + | | 32.7 | 87.2 | 13.9 | 408 | - | - | - | - |
| 13 | 20-24 | 500 | - | | 33.5 | 83.1 | 12.4 | 457 | 3465 | 39 | 25 | 19 |
| 14 | 14 | 600 | - | | 38.8 | 75.5 | 15.1 | 445 | 3255 | 38 | 26 | 21 |
| 15 | 12-13 | 1000 | - | | 17.2 | 76.3 | 13.5 | 436 | 3535 | 39 | 28 | 21 |
| 16 | 9 | 500 | + | | 48.8 | 84.3 | 14.6 | 395 | 3965 | 40 | 23 | 26 |
| 17 | >30 | 500 | + | | 41.7 | 75.4 | 12.4 | 425 | 3620 | 38 | 20 | 20 |
| 18 | 15-19  20-24 | 1200  1200 | - | | 29.2 | 62.0 | 14.3 | 379 | 4005 | 40 | 30 | - |
| 19 | 9 | 600 | + | | 35.8 | 71.3 | 11.6 | 376 | 3105 | 38 | 27 | 31 |
| 20 | >30 | 500 | + | | 41.0 | 83.3 | 13.6 | 378 | 3865 | 39 | 31 | 20 |
| 21 | - | 500 | + | | 25.4 | 63.9 | 15.1 | 366 | 3940 | 40 | 28 | 22 |
|  | = antifungal medicine use in MPW ± 2 weeks | | | | | | | |  |  |  |  |
|  | | | | | | | | | | | | |
| Table 1 continued. Use of antifungal vaginal tablets (active ingredients clotrimazole or miconazole) and oral fluconazole in relation to anogenital distance (AGDas, AGDap) and penile width measurements among 25 mother/boy pairs. | | | | | | | | | | | | |
| **Fluconazole oral tablet use** | | | | | | | | | | | | |
| **ID** | **Weeks** | **Doses** |  | | **AGDas (mm)** mean | **AGDap (mm)** mean | **Penile width (mm)** mean | **Days since conception** | **Birth weight (g)** | **Gestational age (weeks)**  **at birth** | **Maternal age (y)** | **Maternal BMI** |
| 22 | 10-14 | 150 |  | | 22.8 | 57.6 | 13.8 | 361 | 4010 | 41 | 29 | 24 |
| 23 | 10-14 | 150 |  | | 29.8 | 68.8 | 15.6 | 377 | 3890 | 39 | 26 | 22 |
| 24 | 6 | 150 |  | | 32.7 | 69.0 | 15.0 | 380 | 4640 | 41 | 33 | 22 |
| 25 | 15-19 | 150 |  | | 29.5 | 67.3 | 13.0 | 381 | 2575 | 36 | 30 | 17 |
|  | = antifungal medicine use in MPW ± 2 weeks | | | | | | | |  |  |  |  |

Table 2 Mean (SD) penile width in mm in boys at three months according to maternal use of antifungal medicine in pregnancy. Beta values represent adjusted mean difference (95% confidence intervals, 95% CI) in penile width in boys whose mothers used antifungal medicine in pregnancy, and non-users.

|  |  | **Penile Width** | | |
| --- | --- | --- | --- | --- |
|  | N | Mean (SD) mm | β^a^  mm | 95% CI |
| **No antifungal medicine use** (reference) | 715 | 13.5 (1.3) | Reference | |
| **Vaginal tablets^b^** | 21 | 13.5 (1.0) | -0.2 | (-0.7; 0.4) |
| Only vaginal tablets^c^ | 11 | 13.3 (1.0) | -0.2 | (-0.9; 0.6) |
| Vaginal tablets in combination with cream^c^ | 10 | 13.6 (1.1) | -0.1 | (-0.9; 0.7) |
| Vaginal tablets *in* masculinisation programming window^d^ | 11 | 13.6 (1.1) | -0.1 | (-0.8; 0.7) |
| Vaginal tablets *outside* the masculinisation programming window^d^ | 10 | 13.4 (1.1) | -0.2 | (-1.0; 0.5) |
| **Vaginal cream^b^ only** | 23 | 13.9 (1.1) | 0.4 | (-0.2; 0.9) |
| **Fluconazole oral tablets** | 4 | 14.3 (1.2) | 0.4 | (-0.2; 0.9) |
| **Use of antifungal medicine but type not specified** | 39 | **13.1 (1.2)** | **-0.5** | **(-0.9; -0.1)** |
| ^a^ Adjusted for age and z-score for weight  ^b^ Active ingredients miconazole or clotrimazole  ^c^ Sub analysis where antifungal vaginal tablet use (N= 21) has been divided into “only vaginal tablet use” and “vaginal tablet used in combination with cream”  ^d^ Sub analysis where antifungal vaginal tablet use (N= 21) has been divided into “ Vaginal tablets *in* masculinization programming window” and “ Vaginal tablets *outside* the masculinization programming window” | | | | |
